# Spatial control of perilacunar canalicular remodeling during lactation

**DOI:** 10.1038/s41598-024-63645-0

**Published:** 2024-06-25

**Authors:** Michael Sieverts, Cristal Yee, Minali Nemani, Dilworth Y. Parkinson, Tamara Alliston, Claire Acevedo

**Affiliations:** 1 Department of Mechanical Engineering, University of Utah, Salt Lake City, UT 84112 USA; 2 Department of Orthopedic Surgery, University of California, San Francisco, CA 94131 USA; 3 Advanced Light Source, Lawrence Berkeley Laboratory, Berkeley, CA 94720 USA; 4 Department of Biomedical Engineering, University of Utah, Salt Lake City, UT 84112 USA; 5 Department of Mechanical and Aerospace Engineering, University of California San Diego, San Diego, CA 92161 USA

**Keywords:** Targeted bone remodelling, Bone, X-ray tomography, Osteocytes, Image processing

## Abstract

Osteocytes locally remodel their surrounding tissue through perilacunar canalicular remodeling (PLR). During lactation, osteocytes remove minerals to satisfy the metabolic demand, resulting in increased lacunar volume, quantifiable with synchrotron X-ray radiation micro-tomography (SRµCT). Although the effects of lactation on PLR are well-studied, it remains unclear whether PLR occurs uniformly throughout the bone and what mechanisms prevent PLR from undermining bone quality. We used SRµCT imaging to conduct an in-depth spatial analysis of the impact of lactation and osteocyte-intrinsic MMP13 deletion on PLR in murine bone. We found larger lacunae undergoing PLR are located near canals in the mid-cortex or endosteum. We show lactation-induced hypomineralization occurs 14 µm away from lacunar edges, past a hypermineralized barrier. Our findings reveal that osteocyte-intrinsic MMP13 is crucial for lactation-induced PLR near lacunae in the mid-cortex but not for whole-bone resorption. This research highlights the spatial control of PLR on mineral distribution during lactation.

## Introduction

Lactation exerts one of the largest metabolic demands a mother will ever experience^1^. To support lactation, there is a particular need for calcium and phosphorous. When nursing, humans will secrete 300–400 mg of calcium into milk each day from various sources, including diet, urine, and bone^2^. The skeleton is the primary source of calcium for lactation^2–4^. During lactation, the maternal skeleton is resorbed to provide calcium for breast milk^2–4^. This bone resorption significantly reduces skeletal mass. Humans’ skeletal mass will be reduced by approximately 5–8%, and rodents’ will be reduced by approximately 20–30%^2^. Bones must balance the competing demands of their mechanical and metabolic functions throughout this process^5^. For instance, during lactation, there is an active contest between quickly releasing calcium and maintaining the integrity of the bone’s extracellular matrix. Osteocytes are essential in directing this remodeling process and balancing these demands^5^.

Osteocytes are the most abundant bone cells. They form from mature osteoblasts encased by bone and housed in small voids called lacunae and connect through a dense network of dendritic branches through narrow channels called canaliculi^6^. Osteocytes utilize this network to coordinate remodeling throughout the bone^4,7,8^. During lactation, osteocytes recruit and direct osteoclasts to perform whole-bone resorption to mobilize minerals^9,10^. Osteoclasts’ primary role is to resorb bone by secreting acid and proteases^10^. There is increased osteoclast activity and resorption during lactation^11^. In cortical bone, lactation-induced resorption occurs on the endocortical surface of the bone^12–14^. This resorption has been associated with reduced whole-bone stiffness^12,14,15^. Osteoclast resorption of the maternal skeleton is one of the clear mechanisms used to satisfy the demands of lactation^9,11^. Another approach to meet the mineral demands of lactation involves osteocytes resorbing bone in their proximity^9^.

Osteocytes are multi-functional cells that directly remodel their surroundings through perilacunar/canalicular remodeling (PLR)^2,4,7,8,12,16–18^. During PLR, an osteocyte resorbs the perilacunar/canalicular extracellular matrix (ECM) by releasing enzymatic proteins, such as matrix metalloproteases 13 (MMP13). We have previously shown that MMP13 is essential for PLR^16^, emphasizing that MMP13 helps to maintain bone homeostasis and quality. During metabolic stress, such as lactation, osteocytes stimulate PLR to meet the calcium demands of milk, as observed with enlarged lacunae volume and upregulation of PLR-related gene expression, including MMP13^4^. Since MMP13 is required for PLR activity, we investigate how osteocyte-intrinsic MMP13 regulates PLR during lactation.

PLR remodeling controls mineral content in the expansive lacunar canalicular network (LCN). Due to the vast surface area of the LCN within the bone, 215 m^2^ in the average adult^19^, small amounts of remodeling in the network can mobilize large amounts of minerals. Liu et al. stated, “PLR likely serves as the key controller to regulate skeletal homeostasis by optimizing the balance between mineral resorption and mechanical integrity of the maternal skeleton^5^.” As the mineral surrounding the lacunae is removed, lacunar volume increases, which impacts bone’s mechanical properties^12^. Kaya et al. found that increased lacunar volume caused local reductions in bone’s elastic modulus^12^. Because of the link between lactation and PLR, lactation models are used to induce PLR and study its impact on the bone’s microstructure. One effective technique to study the effect of PLR on the microstructure is synchrotron X-ray radiation micro-computed tomography (SRµCT).

SRµCT provides 3D reconstructions of the bone’s microstructure that are used to measure the size and arrangement of different features within the bone, such as the osteocyte lacunae^20,21^. Additionally, SRµCT is a densitometry technique, meaning the images provide information on the bone’s tissue mineral density (TMD) or mineralization. Combining SRµCT images with analysis techniques can provide insight into local changes within the bone, such as the arrangement of vascular canals, the osteocyte lacunae, and the local mineralization surrounding the osteocyte lacunae. However, SRµCT is limited by phase contrast at material interfaces. Phase contrast is an imaging artifact that can result in artificially high-density readings near voids within the bone, which inhibits local density measurements. However, incorporating phase retrieval during image reconstruction to retrieve the absorption portion of the image can minimize the impact of phase contrast, allowing for local measurements^22–24^. SRµCT imaging produces valuable data that can be used to gain new insight into the role and regulation of PLR.

Recently, techniques such as SRµCT and synchrotron radiation nano-CT have been used to gather information about the spatial distribution of microstructural features and mineralization within the bone. Some research has been done regarding the arrangement of the vascular canals and the osteocyte lacunae throughout the bone^25,26^. Trend et al. studied the arrangement of these features and found that larger lacunae tend to be in densely vascularized regions of cortical bone^26^. They suggest that there could be a coupling mechanism between the bone’s vasculature and the osteocyte lacunae, which regulates the lacunar volume. Other research using nano-CT has investigated how the bone’s mineral is arranged with respect to the lacunar canalicular system (LCS), considered as the smallest level of vascularization in bone^13,27–29^. There is some disagreement between studies on the spatial relationship of minerals with respect to the LCS^13^. Generally, a hypermineralized region of tissue is reported immediately surrounding the lacunae. At further distances, this hypermineralized matrix decreases until the mineral concentration reaches asymptotic values. It is expected that heterogeneity caused by this variation in mineralization contributes to bone’s resistance to fracture by increasing the amount of energy bone can dissipate^30^. Combining these spatial measurements with a lactation model known to induce PLR provides an opportunity to study the spatial effects of PLR. Particularly, we are interested in answering the questions: how does lactation impact the spatial arrangement of osteocyte lacunae and mineralization surrounding the lacunae, and what role does osteocyte-intrinsic MMP13 have in these spatial changes?

PLR plays a critical role in bone physiology, ensuring the efficient exchange of minerals between the osteocytes and the surrounding environment. However, the specific mechanisms underlying PLR, particularly the sites and pathways of mineral resorption and transport, remain poorly understood. Understanding the spatial response of PLR during the high metabolic demand of lactation can further elucidate how osteocytes balance metabolic and mechanical demands within the bone matrix. In this study, we seek to address this knowledge gap by investigating the spatial changes in bone’s lacunar volume and mineralization at the macro- and microscales through SRµCT. Additionally, by leveraging phase retrieval techniques, we can accurately measure local changes in bone matrix mineralization around thousands of osteocytes. We anticipate gaining valuable insights into the resorption and transport of minerals from the lacuna-canaliculi system to the bloodstream, elucidating the multi-scale pathways involved in calcium delivery. We hypothesize that PLR is more active in lacunae near the bone’s vasculature. We propose the existence of a natural mineral barrier consistently positioned at a specific distance from the lacunae, restricting the expansion of lacunar volume. Additionally, we aim to investigate the role of MMP13 in the spatiotemporal release of minerals and its impact on the integrity of the lacunae. Understanding the mechanisms of mineral regulation that rapidly deliver calcium and maintain bone quality near the osteocyte is instrumental in developing bone fragility treatments that target osteocytes to restore bone quality.

## Results

### Osteocyte-intrinsic MMP13 is dispensable for lactation-induced bone loss

To elucidate the role of osteocyte-intrinsic MMP13 in PLR during lactation, we use an established mouse line where floxed MMP13 allele^31^ is ablated under the 9.6-kb DMP1-Cre promoter^32^, resulting in osteocyte-targeted MMP13 deletion (MMP13^ocy−/−^)^7^. Reduction of MMP13 mRNA and protein expression in osteocytes of MMP13^ocy−/−^ female mice, relative to age-matched Cre-negative female controls^33^, was confirmed by immunofluorescence (IF) and qRT-PCR of genes related to PLR (Figs. S1, S2).

**Figure 1:**
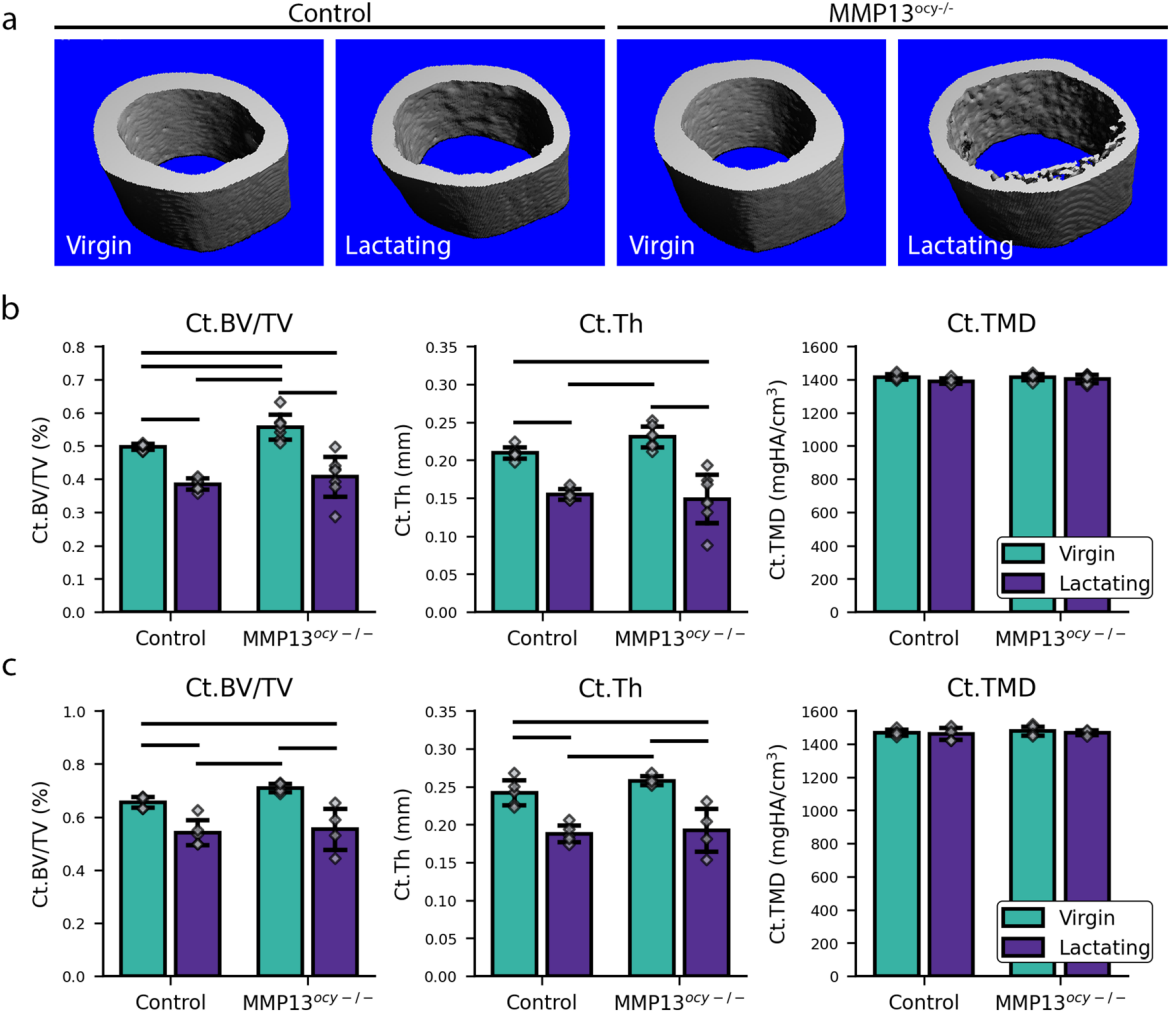
Lactation reduces the whole-bone volume in control and osteocyte-intrinsic MMP13 deficient groups indicative that osteocyte-intrinsic MMP13 is dispensable for lactation-induced changes in cortical bone geometry (**a**) Representative standard µCT images of mid-diaphysis femoral cortical bone. (**b**) Measurements of femoral cortical (Ct.) bone volume fraction (BV/TV), Thickness (Th), and Tissue Mineral Density (TMD) (N = 7/group) using µCT of mouse femurs. (**c**) Measurements of tibia cortical bone using SRµCT (N = 4–5/group). Significant differences (p < 0.05) are denoted with a horizontal line between groups. All bar-chart data are represented by mean ± standard deviation. Individual data points are also displayed on the bar charts.

The effect of lactation on female MMP13^ocy−/−^ mice was compared to female controls^33^ in this well-established model of PLR induction. Tissues were collected following two weeks of lactation when the mice were 16 weeks old. Cortical bone geometry and microstructure were examined using µCT of mouse femurs and using SRµCT of mouse tibia. As expected, both methods provide strong evidence that lactation alters whole-bone geometry and structure by reducing bone volume fraction and cortical thickness resorbed from the endosteal side (Fig. 1), confirming the rigor of this lactation model and our outcome measures. Specifically, in the control mice, lactation resulted in a significant reduction of cortical thickness (22–26%) and bone volume fraction (18–23%). Osteocyte-intrinsic deletion of MMP13 does not significantly diminish the effects of lactation on the whole-bone geometry. Similar to control mice, MMP13^ocy−/−^ mice show lactation-induced reductions in cortical thickness (26–36%) and bone volume fraction (22–27%) across tibia and femur cortical bone parameters (Fig. 1). Therefore, osteocyte-intrinsic MMP13 is not required for lactation-induced cortical bone loss via resorption of the endosteum^4,14,34^ (Fig. S3).

### Lactation-induced increase of lacunar volume requires osteocyte-intrinsic MMP13

**Figure 2:**
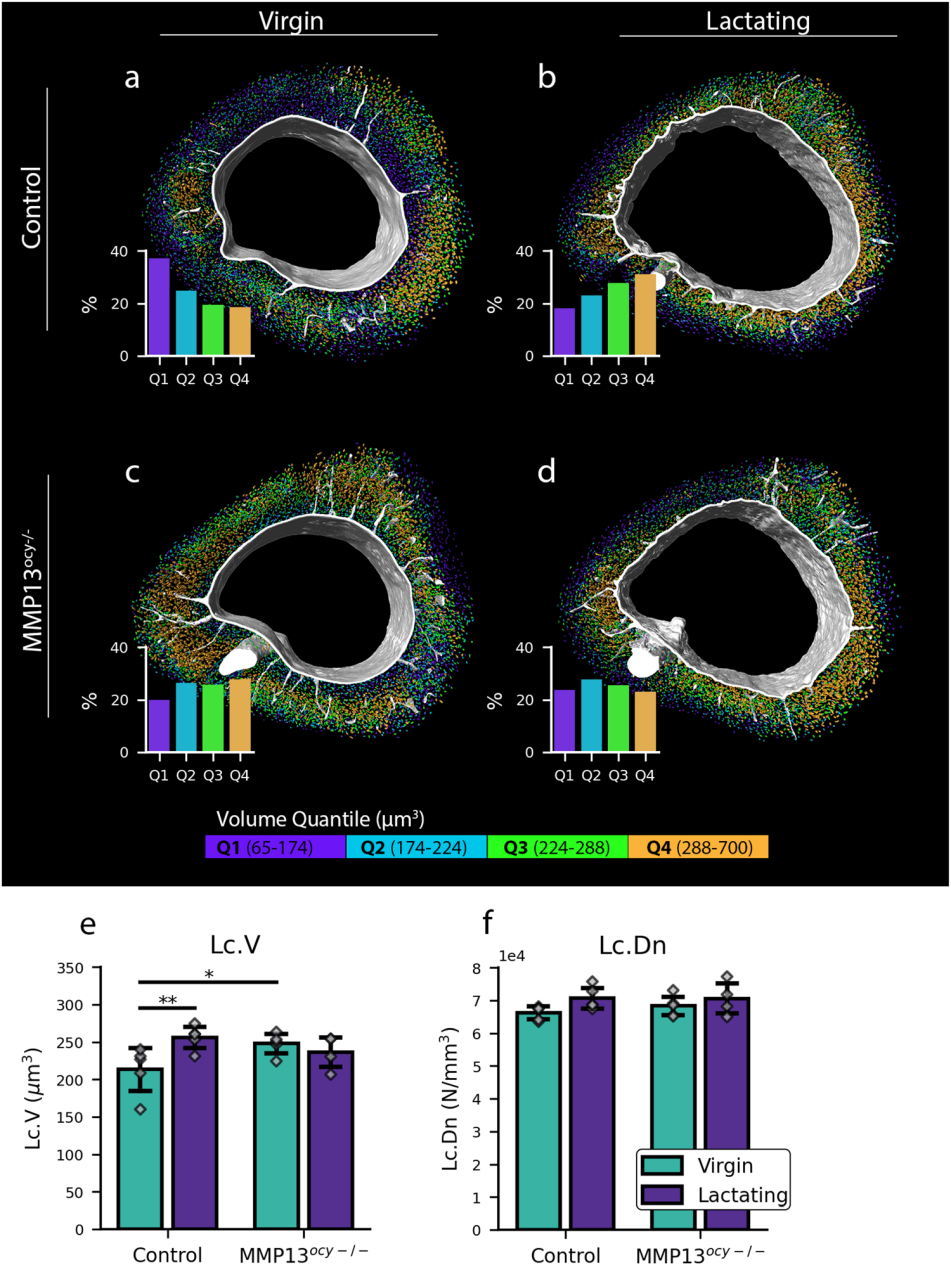
Lactation causes increased lacunar volume in the control groups. Osteocyte-intrinsic MMP13 deficiency prevents lacunar resorption. (**a**–**d**) Representative 3D renderings of the bone’s lacunae and vasculature for each group. The vasculature is colored white, and the lacunae are colored according to their volume quantile. Each image is accompanied by a bar chart showing the proportion of lacunae in each quantile for each group. (**e**,**f**) Bar plots for the lacunae measurements lacunar volume (Lc.V) and lacunar density (Lc.Dn) represented by mean ± standard deviation. The individual data points are also included with the bar plots (N = 4–5). Significant differences are denoted with a horizontal line between groups on the bar chart.

Bone from lactating control mice exhibits a substantial increase in lacunar volume, measuring approximately 25% larger compared to the virgin control group (Fig. 2e). Other researchers have observed this phenomenon and attributed it to increased PLR in response to lactation^4,7^. No difference in lacunar volume was observed between the virgin and lactating MMP13^ocy−/−^ groups (Fig. 2e), indicating that osteocyte-intrinsic MMP13 deficiency prevents calcium resorption from lactation in lacunae. This is consistent with prior research showing that MMP13 is an important PLR enzyme^16^. Therefore, although osteocytic MMP13 is dispensable for lactation-induced loss of cortical bone volume (acting on the endosteal side) at the macroscale, it is essential for the lactation-induced increase in lacunar volume at the microscale.

Analyzing the distribution of lacunae in volume quantiles further illustrates the PLR response. All lacunae for bones in all groups were divided into four quartiles based on volume (Q1–Q4), where Q1 contains the 25% of lacunae with the smallest volume, and Q4 contains the 25% of lacunae with the largest volume (Fig. 2a–d, Supplementary Movie 1). In the control virgin group, most lacunae (37%) are in Q1, and the least lacunae (19%) are in Q4 (Fig. 2a). We observe the opposite trend in the control lactating group (Fig. 2b), indicative that osteocyte lacunae are the largest with lactation. MMP13^ocy−/−^ bone neither exhibits this trend in the distribution of lacunar size nor the lactation-induced shift in lacunar size (Fig. 2c,d). Therefore, osteocytic MMP13 participates in establishing homeostatic osteocyte lacunar size and calibrating lacunar size in response to lactation.

Lacunar density remains consistent despite the reduction of cortical bone during lactation (Fig. 2f). We found that the number of lacunae per bone volume wasn’t significantly affected even with the significant lactation-induced reduction in bone mass and cortical thickness. This suggests that lacunae are approximately uniformly distributed throughout the bone.

### Lactation-induced PLR is most likely to occur near the vascular canals or the endosteum

**Figure 3:**
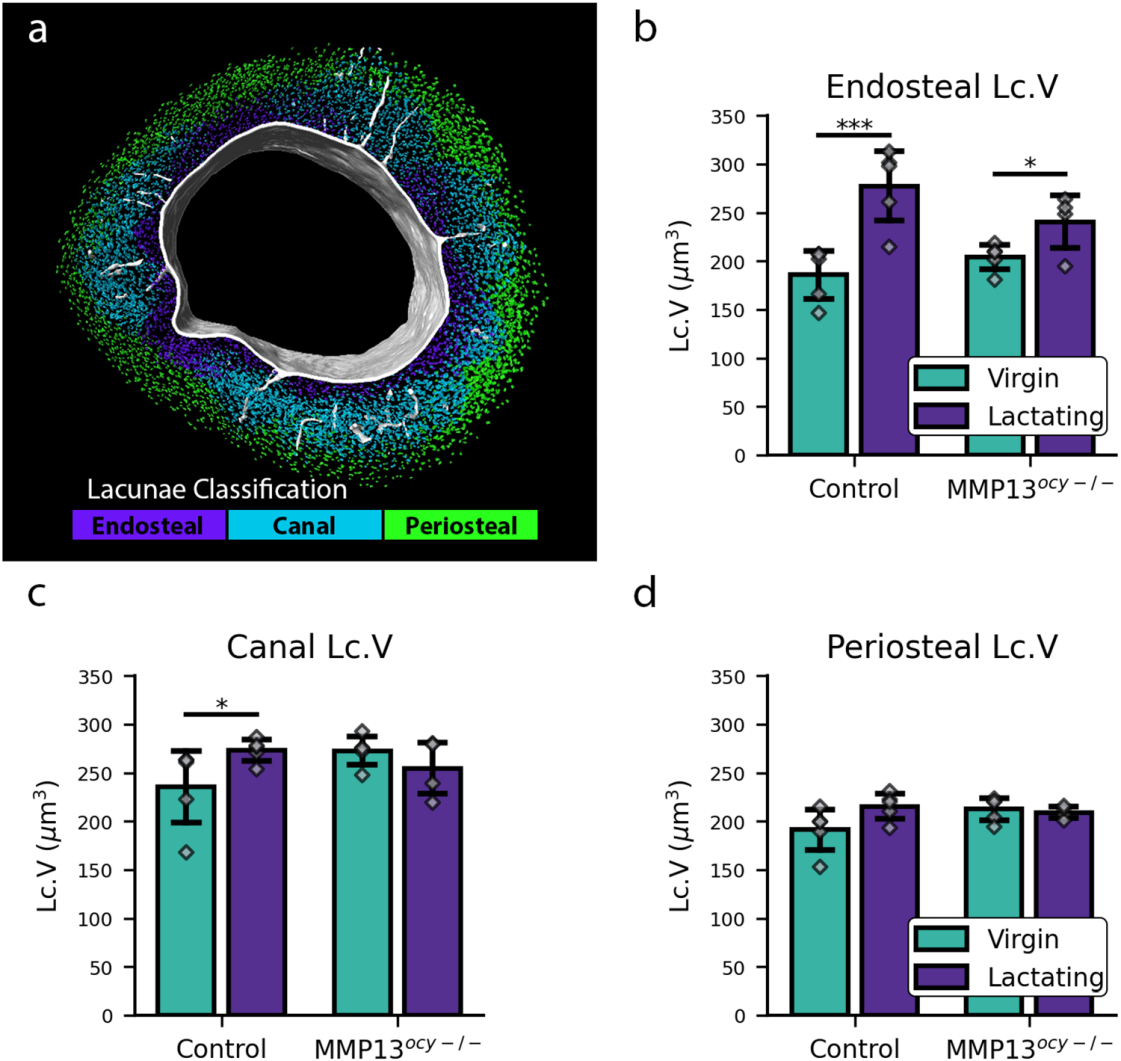
Spatial control of lactation induced changes in lacunar volume. (**a**) Example image displaying lacunae assigned to the closest vascular surface (endosteum, canals, periosteum). (**b**–**d**) Lacunar volume measurements for lacunae that are assigned to each surface (N = 4–5). Significant differences are denoted with a horizontal line between groups. All bar-chart data are represented by mean ± standard deviation. The bar charts also display individual data points.

The images in Fig. 2 show that lactation induces changes in lacunar volume spatially heterogeneously. This heterogeneous change in lacunar volume could suggest that PLR is more likely to occur in certain regions of the bone. To test the hypothesis that some populations of osteocytes are more responsive to lactation than others and that those differences correspond to osteocytic proximity to vascular interfaces, we modeled the distance of each osteocyte lacunae to the cortical bone vasculature and the endosteal and periosteal surfaces to see which showed the greatest lactation-induced changes in lacunar size. Each lacuna was assigned to the endosteum, canals, or periosteum based on which surface it was closest to (Fig. 3a). The lacunar volume for each sub-group was analyzed.

We found that lactation causes a significant increase in lacunar volume for lacunae near the endosteum or the canals in the control group (Fig. 3). In the endosteal sub-group, lacunar volume increases significantly with lactation regardless of genotype. However, the magnitude of this increase is particularly prominent in the control group, exhibiting a remarkable 49% volume increase. Conversely, the MMP13 deficient group experiences a comparatively smaller increase of 18% in lacunar volume during lactation. In the canal sub-group, the lacunar volume only increased significantly with lactation in the control group. This indicates that lactation induces PLR near densely vascularized regions of cortical bone. In the periosteal sub-group, there was no significant increase in volume in either genotype, indicating that PLR is less active in the periosteal region.

**Figure 4:**
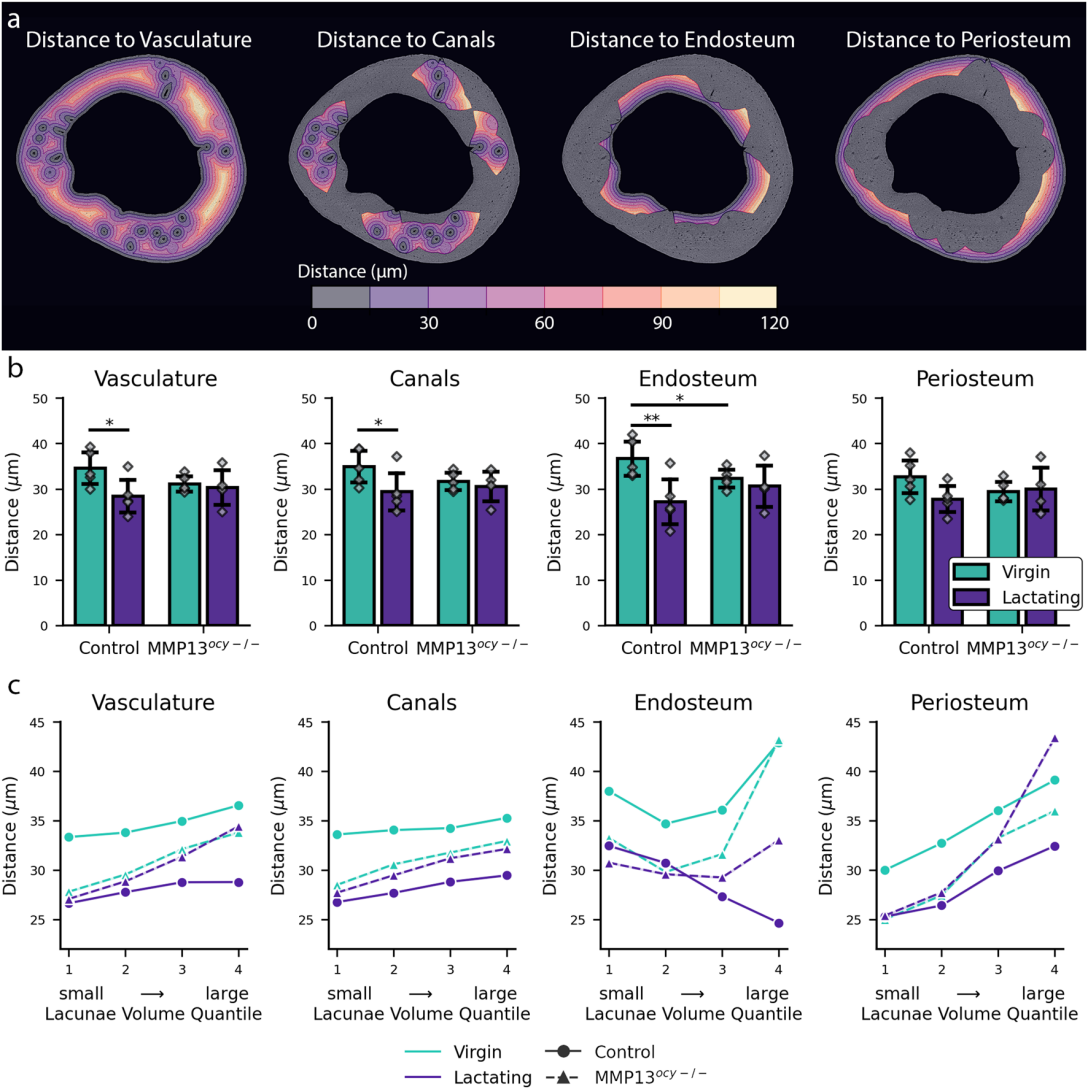
Lacunae tend to be closer to the bone’s vasculature during lactation. (**a**) 2D slices of the 3D distance map to the bone’s vasculature, canals, endosteum, and periosteum. (**b**) Bar plots describing the mean minimum distance from the bone’s lacunae to the bone’s features (N = 4–5). (**c**) Line plot showing the mean minimum distance to bone’s features for lacunae in each volume quantile (N = 4–5). All bar-chart data are represented by mean ± standard deviation. Individual data points are also displayed on the bar charts. Significant differences (p < 0.05) are denoted with a horizontal line between groups.

Lactation caused the lacunae to be significantly closer to the vasculature in the control group (Fig. 4b). To investigate the lacunar proximity to the vasculature, we measured the minimum distance of each lacuna to the nearest vascular surface (Fig. 4a). The bone’s vasculature was defined as the bone’s canals combined with the bone’s endosteal and periosteal surfaces (Fig. 4a). This definition was selected to analyze interfaces where the transport of liberated minerals into the systemic circulation could occur. Additionally, we considered the vascular canals and endosteal and periosteal surfaces separately (Fig. 4a). This approach showed that lacunae from the lactating control group were significantly closer to the bone’s vasculature (Fig. 4b). No significant differences were observed with lactation in the MMP13 deficient group (Fig. 4b). These results illustrate that lactation causes lacunae to be closer to the bone’s vasculature and that the MMP13 deficiency disrupts this behavior.

To further investigate the relationship between lacunar volume and proximity to the vasculature, we analyzed the mean minimum distance for lacunae in different volume quantiles (Fig. 4c). Analyzing the distance to the vasculature, slight variations were observed with each volume quantile. The lacunae in the lactating control group were closest to the vasculature in all volume quantiles. The control virgin and lactating groups displayed similar behavior, except the lactating group was offset at lower distances (Fig. 4c). The MMP13 deficient virgin and lactating groups exhibited similar behavior without any offset (Fig. 4c). The canal group of lacunae showed very similar results to the overall vascular results. In the endosteum group, lacunae from the control lactating group showed a trend for larger lacunae to be closer to the endosteum. This trend was not observed in any other group (Fig. 4). The periosteum group of lacunae shows a trend for larger lacunae to be further from the periosteum in all groups. These results further confirm that the canals and the endosteum surface are preferred regions for lactation-induced PLR in the control group.

### Lactation results in hypomineralization regions within the bone

**Figure 5:**
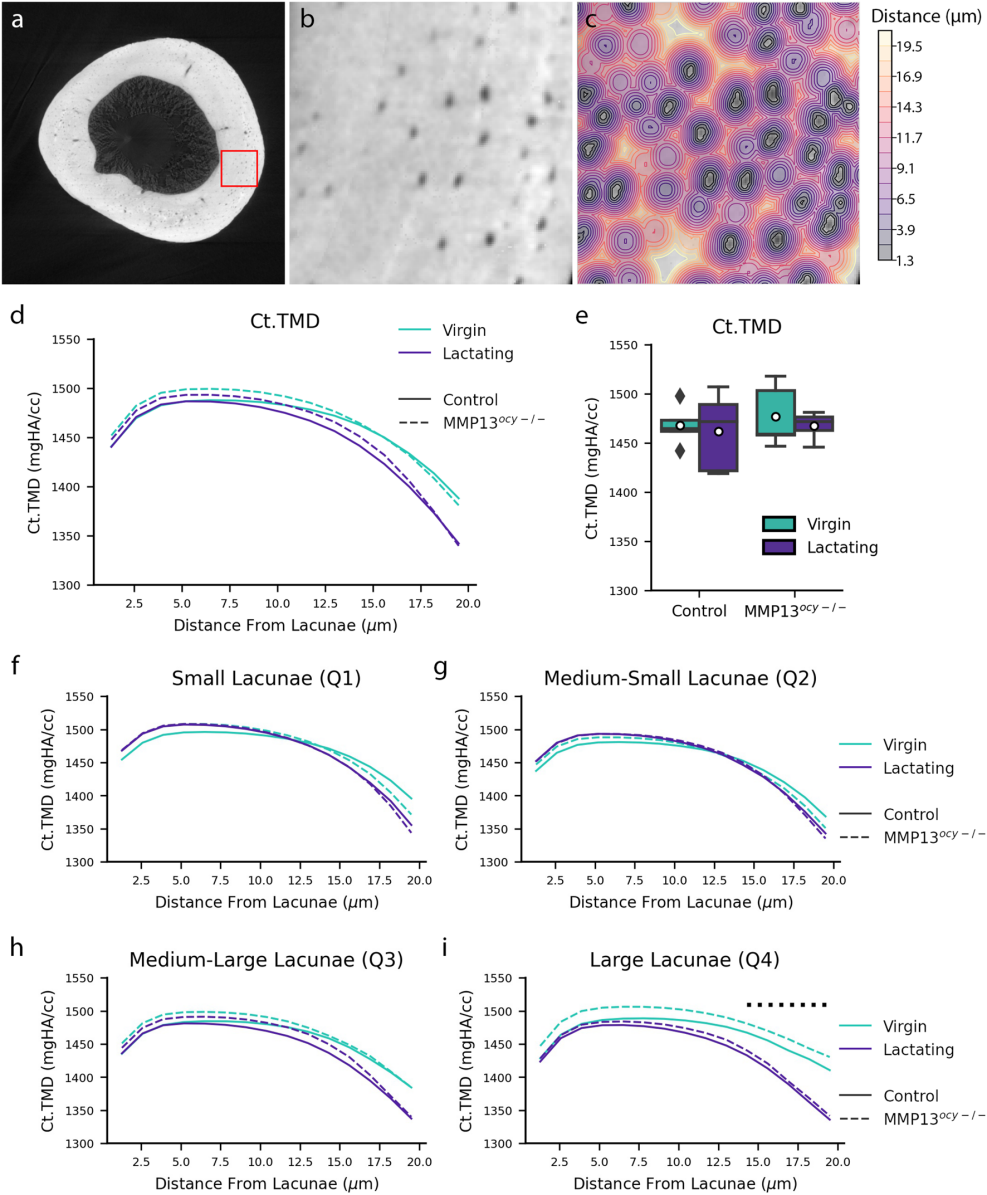
Lactation results in hypomineralized regions of bone near large lacunae. (**a**–**c**) Images illustrating how the mineralization profiles were constructed. Left to right, these images show a 2D SRµCT image of bone with a red callout box, the highlighted region of bone zoomed in to see the lacunae, and the distance map of the lacunae in the bone. (**d**) A line plot shows the overall mineralization profile surrounding the lacunae (N = 4–5). (**e**) A boxplot shows each group’s overall distribution of mineralization (N = 4–5). (**f**–**i**) Line plots showing the mineralization profiles surrounding lacunae in each volume quantile. Quantile 1 contains the smallest lacunae, and quantile 4 contains the largest lacunae (N = 4–5). Significant differences between the virgin and lactating groups on the line plots are shown with a horizontal dashed line above the profiles.

To test the hypothesis that a mineral barrier surrounding the lacunae may protect the bone from rampant resorption, we examined the spatial variation of mineralization with respect to the location of the lacunae. We constructed mineralization profiles for each bone using a distance map from the edge of the lacunae SRµCT images (Fig. 5a–c). The SRµCT images were reconstructed with phase retrieval to minimize the impact of phase contrast on the mineralization values (Fig. S4). These profiles show a hypermineralized region of bone within 10 µm from the lacunae’s edge and a more distant hypomineralized region of bone between 12.5 and 20 µm from the lacunae’s edge (Fig. 5d). To determine if local mineralization is impacted by lacunar volume, the spatial relationship of mineralization with respect to the lacunar edge was further analyzed by creating mineralization profiles for lacunae in different volume quantiles (Fig. 5f–i). Despite changes in lacunar size, there is relatively little or no change in the mineral density nearest the lacunar edge (Fig. 5f–i), keeping this mineral boundary intact.

Lactation-induced canalicular resorption is most prevalent in large lacunae, which are expected to have also undergone perilacunar resorption. The mineralization profiles for lacunae of all sizes show a hypomineralized region near 12.5–20 µm from the lacunae’s edge (Fig. 5d). The profiles for the smaller lacunae, Q1 and Q2, were very similar for all groups (Fig. 5f,g). The profiles are still similar in Q3, but the lactating groups start to show reduced mineralization between 14 and 20 µm away from the lacunae (Fig. 5h). Unlike Q1–Q3, the largest lacunae, Q4, show a significant lactation-dependent reduction in mineralization between 14 and 20 µm away from the lacunae (Fig. 5i). This lactation-induced reduction in mineral 14–20 µm away from the lacunae could result from canalicular resorption of mineral. MMP13 has little effect on the lacunae’s mineral boundary or lactation-induced canalicular resorption.

## Discussion

This study provides evidence of spatial control of PLR and mineral homeostasis during lactation. We identified three measurements that indicate this spatial control exists. First, lactation causes an increase in lacunar volume in lacunae situated near the endosteum and canals but not near the periosteum (Fig. 3). Lactation also resulted in lacunae being closer to the bone’s vasculature (Fig. 4). Finally, large lacunae tend to have a surrounding hypomineralized region of bone in the lactating groups compared to the virgin bone (Fig. 5). These measurements suggest that PLR has a spatial relationship to the bone’s vasculature, and mineral homeostasis is related to lacunar volume. Osteocytic MMP13 is required for lactation-induced PLR near intracortical bone vasculature but not for remodeling near the endosteum. Additionally, we found that during lactation, the osteocyte-specific deletion of MMP13 inhibited PLR but still allowed osteoclasts to perform whole-bone resorption, evident through lactation-dependent loss of bone volume and cortical thickness (Figs. 1, 2).

One example of the spatial control of PLR during lactation is that lactation-induced PLR is more evident in regions of the bone near the endosteum or vascular canals (Fig. 3). In the endosteal region of the bone, lactation induced a drastic increase in lacunar volume in the control group and a sizable increase in the MMP13^ocy−/−^ group. Given our finding that the deletion of osteocyte-intrinsic MMP13 prevents lacunar changes (Fig. 2), it is plausible that the increase in lacunar volume in the endosteum region may be related to osteoclasts’ whole-bone resorption on the endocortical surface^12–14^ (Fig. 1). This whole-bone resorption would require systemic MMP13^16^, and MMP13 may reach some lacunae via diffusion through the LCN. This could explain the significant increase in lacunar volume in the endosteal region with lactation in the MMP13^ocy−/−^ group. In the region of the bone near the canals, lactation induced a significant increase in lacunar volume in the control group, while no difference was observed in the MMP13^ocy−/−^ group. Additionally, the canal region of the bone generally contained larger lacunae than the other regions, which is consistent with what Trend et al. observed^26^. Their study found that large lacunae were associated with densely vascularized regions of the bone and suggested that could be due to an osteo/angiogenic coupling mechanism because this relationship was not observed in bones deficient in vascular endothelial growth factor (VEGF)^26^. No lactation-induced changes in lacunar volume were observed in the periosteum region of the bone. This could be to preserve the mechanical properties of the bone. The periosteal surface of the bone resists more bending than bone closer to the endosteum^35,36^. PLR results in larger lacunae, which Kaya et al. showed to have reduced local elastic modulus^12^. One hypothesis is that the osteocytes’ mechanosensing may preserve the volume of these lacunae to maintain the bone’s resistance to bending during lactation. These findings support that lactation-induced PLR has a spatial preference.

The trend for lacunae from control lactating bone to be near the bone’s vasculature could be evidence of the optimization of bone resorption and PLR. Since the mineral mobilized by PLR must then be transported to satisfy lactation’s metabolic demand, the mineral’s delivery would be more efficient if it was required to travel less distance. The proximity to the vasculature in the control lactating group is described by lacunae being significantly closer to the canals and the endosteum (Fig. 4b). Some of these changes in vascular proximity could be attributed to changes in geometry due to whole-bone resorption. However, in the endosteal region, the control lactating group shows a trend that the larger lacunae are closer to the endosteum than the smaller lacunae (Fig. 4c). This suggests that in this region, increased PLR occurs near the endosteum. This spatially targeted PLR response further elucidates how bone optimally responds to stimuli and demands.

The mineralization profiles further demonstrate bone’s spatial heterogeneity (Fig. 5). Similar to other groups, we observed a hypermineralized region of bone immediately surrounding the lacunae, which decreased at further distances^27,28^. In this study, most lacunae have similar mineralization profiles between the virgin and the lactating groups (Fig. 5d,f–h). This agrees with Kaya et al.’s finding that there was no difference in normalized mineral profiles surrounding the canaliculi due to lactation^12^. However, here we identified a hypomineralized region 12–20 µm away from the edge of large lacunae due to lactation (Fig. 5i). The hypomineralized region of bone we found suggests that lactation causes minerals to be mobilized through the LCS some distance from the lacunae. One explanation for this resorption of minerals could be peri-canalicular remodeling. Nango et al. found that mineral dissolution begins at the canaliculi, not the lacunae, and that peri-canalicular dissolution occurred independently of peri-lacunar dissolution^12,27^. Additionally, Nango et al. observed that peri-lacunar hypermineralization could persist during peri-canalicular dissolution^27^. This could explain the consistent mineralization values immediately surrounding the lacunae, even surrounding the large lacunae that show hypomineralization in response to lactation. The hypermineralized region immediately surrounding the lacunae, which persists regardless of genotype or lactation, could indicate a method of osteocytes preserving the structure of the LCN. These findings build upon the observations by Nango et al. by illustrating that peri-lacunar hypermineralization persists during lactation even when there is evidence of peri-canalicular dissolution^27^. These mineralization results illustrate bone’s spatial control of minerals even during high metabolic demand. This suggests that distinct regulatory mechanisms control perilacunar remodeling relative to pericanalicular remodeling.

In this work, we found that osteocytic MMP13 is required for PLR during lactation. The expected increase in lacunar volume due to lactation was not observed in animals with an osteocyte-intrinsic deficiency in MMP13 (Fig. 2). This suppression of PLR is expected because MMP13 is a protease that breaks down collagen and other extracellular proteins in the bone matrix, an essential process in resorbing bone^16^. Similar to our results, Tang et al. observed no significant increase in cortical porosity in response to lactation in mice with a systemic ablation of MMP13^16^. A key difference between our study and the previous study by Tang et al. is that Tang et al. used a systemic MMP13 deletion in mice, whereas we used an osteocyte-specific deletion in mice. The systemic deletion of MMP13 resulted in no significant change in bone area in response to lactation^16^. However, we observed significant bone volume and thickness reductions in response to lactation (Fig. 1). These contrasting results suggest that osteoclast resorption requires systemic MMP13; however, osteocyte regulation of osteoclast resorption may proceed despite the osteocyte-specific deficiency of MMP13. It is possible that the inability of MMP13-deficient osteocytes to meet the metabolic demand mineral in lactation leads to a compensatory increase in osteoclast resorption on the endosteal surface^7^. This possibility is supported by the strong induction of TRAP and CatK mRNA expression in the bone from lactating MMP13^ocy−/−^ mice relative to other conditions (Fig. S1).

One limitation of this study is the relatively large voxel size, which causes us to resolve less detail than other studies focused on local mineralization. Other studies on local mineralization have used techniques such as nano CT or transmission electron microscopy, which achieve finer resolution. For example, our images have a voxel size of 1.3 µm while Hesse et al. used a voxel size of 0.05 µm^28^. This reduced resolution doesn’t allow for analysis of the bone’s canaliculi, so we only analyze mineralization with respect to the bone’s lacunae. Due to the large voxel size, some partial volume voxels may contribute to lower TMD values immediately surrounding the lacunae, approximately 0–2.6 µm from the lacunae edge. However, an advantage to using a lower resolution technique is that we can analyze thousands of lacunae per sample, whereas other studies are limited to approximately 2–30 lacunae per sample. An interesting future study would be correlating the mineralization information between SRµCT and nano-CT in bone. Our results indicate that lactation preferentially causes local changes in bone matrix mineralization in osteocytes near vasculature, which could ultimately inform drug delivery strategies or cellular targets to intervene in osteocyte function. Additional studies would be needed to elucidate mechanisms that selectively induce PLR in osteocytes near blood vessels.

In conclusion, this work provides evidence of bone’s spatial remodeling preferences during lactation. We obtained key measurements that confirm spatially dependent remodeling in the control lactating group. We found that PLR is more likely to occur near the endosteum and vascular canals to optimize the delivery of calcium to the body. We also observed a hypermineralized plateau 5 to 10 µm from the lacuna’s edge, which might act as a barrier for the maximum local lacunar volume. However, despite this barrier, lactation-induced PLR effectively releases calcium and lowers mineralization in the surrounding bone matrix reached by canaliculi located more than 14 µm away from the lacuna’s edge. Additionally, we found that osteocyte-intrinsic MMP13 is essential for lactation-induced PLR near lacunae in the intracortical bone but not for whole-bone resorption in the endosteal region or peri-canalicular resorption. These findings help elucidate how bone balances metabolic and mechanical demands during lactation through spatially targeted PLR.

## Methods

### Mice and lactation study

Mice with osteocyte-specific ablation of MMP13 were previously described by Mazur et al.^7^. Briefly, homozygous MMP13^fl/fl^ mice, on an FVB/N background, containing loxP sites flanking exons 3–5 of the MMP13gene (JAX stock #005710)^31^ are bred with hemizygous DMP1-Cre^+/−^ mice (9.6-kb promoter). Hemizygous DMP1-Cre^+/−^, on a C57BL/6 background, express Cre downstream of the dentin matrix protein 1 (DMP1) promoter/enhancer targeting primarily odontoblasts and osteocytes (JAX stock #023047)^32^. Offspring from crossing MMP13^fl/fl^ and DMP1-Cre^+/−^ generates DMP1-Cre^−/−^; MMP13^fl/fl^ (control) or DMP1-Cre^+/−^; MMP13^fl/fl^ (MMP13^ocy−/−^) mice, which are confirmed with PCR genotyping. Control mice were littermates of MMP13^ocy−/−^ mice.

For this lactation study, female control and MMP13^ocy−/−^ mice were randomly assigned to virgin or lactating groups. Lactation mice were time-mated at ten weeks of age for three days. Pregnant mice delivered pups at approximately 14 weeks of age, and litter size was adjusted to 8–10 pups for each lactating mouse to normalize lactation demand. Mice were euthanized 2 weeks after lactation (16 weeks of age) because previous studies have observed dramatic loss in bone volume beginning at 12 days of lactation^4,37^.

All animal procedures in this study were in accordance with ARRIVE guidelines^38^ and approved by the Institutional Animal Care and Use Committee (IACUC) at the University of California, San Francisco. Mice were housed in a pathogen-free environment with a maintained temperature between 69 and 74 °F, humidity between 30 and 70% 12-h light/dark cycle, and access to water and rodent chow (LabDiet 5058) ad libitum.

All methods were performed in accordance with the ARRIVE guidelines and IACUC regulations.

### Standard micro-computed tomography

Skeletal phenotype analysis was completed using standard microcomputed tomography (µCT) on left femurs harvested from mice at 16 weeks of age that were fixed in 10% neutral buffer formalin (NBF) and stored in 70% EtOH. Trabecular analysis on the femoral metaphysis was performed in a 2-mm region (Table S2), and cortical analysis on the mid-diaphysis was performed in a 1-mm region. All samples, N = 7 mouse/group, were scanned on a Scanco µCT50 scanner (SCANCO Medical AG, Brüttisellen, Switzerland) with an X-ray potential of 55 kVp, current of 109 µA, and voxel size of 10 µm. Thresholding and quantification were performed as previously described^7,39–41^.

### Synchrotron radiation micro-computed tomography

Mice tibia were imaged at the tomography beamline (8.3.2) at the Advanced Light Source at Lawrence Berkeley National Lab. The samples were scanned with an energy of 18 keV. For each scan, 1313 projections were collected over 180 degrees of rotation. The resulting images had a voxel size of 1.3 µm.

The SRµCT images were reconstructed using the gridrec algorithm implemented in the Python package Tomopy^24^. The ring artifact in the scans was reduced using the polar ring removal functionality in Tomopy. Two data reconstructions were used for analysis, one without phase retrieval and one with phase retrieval. The images reconstructed without phase retrieval were used to segment the bone’s microstructural features. These images were used to preserve the maximum resolution for the small microarchitectural features because phase retrieval slightly degrades resolution. The images reconstructed with phase retrieval were used for tissue mineral density values. In this case, phase retrieval was used to retrieve the image’s absorption component. Phase retrieval was performed using Paganin phase retrieval implemented in the phaseCT repository from Forien et al.^22,42^. The absorption ($\beta$) and phase ($\delta$) coefficients for hydroxyapatite used for phase retrieval were determined using the Center for X-ray Optics Database^43^. The $\beta$ and $\delta$ values used were 1.48130805E−08 and 2.01842317E−06, respectively. The effectiveness of the phase retrieval was confirmed through phase simulation using Syris, the synchrotron radiation imaging simulation repository^23^ (Fig. S4).

The microstructural features within the bone were segmented using the SRµCT images. All segmentation was performed using the Dragonfly software, Version 2020.2 for Windows (Object Research Systems (ORS) Inc, Montreal, Canada, 2020). The bone and background were separated using simple thresholding and morphological closing. Some manual segmentation was performed to ensure the background and the bone were segmented successfully. Measurements of the segmented bone were obtained using the Bone Analysis tool in Dragonfly. This tool yielded standard bone µCT measurements of bone volume, bone volume fraction, and cortical thickness^44^.

**Figure 6:**
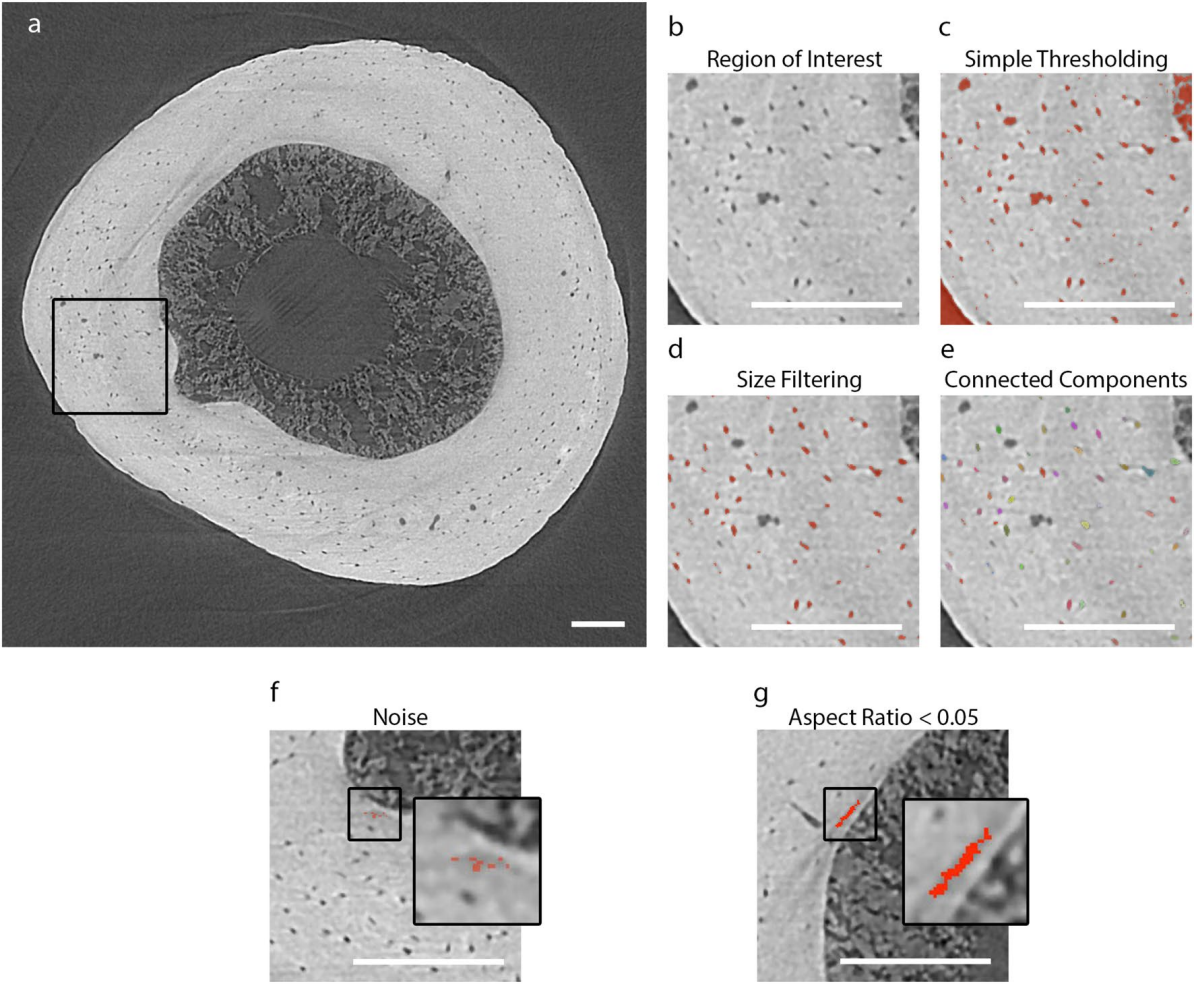
Lacunae segmentation process. (**a**) Overview 2D SRµCT image of bone with a black callout box, which is used as the region of interest to depict the lacunae segmentation steps. (**b**) A zoomed-in image of the region of interest. (**c**) An example of the simple thresholding used to segment all of the voids within the bone. (**d**) An example of the size filtering used to refine the segmentation to the lacunae. (**e**) An example of converting the segmented region into separate connected components of lacunae using a 26-connected approach. This is the segmentation used for further analysis (**f**) An example of noise removed by requiring components to touch the segmentation from the smoothed image. (**g**) An example of a component removed from the segmentation based on its aspect ratio. The aspect ratio requirement removes long, thin components like the one shown here. All white scale bars represent 100 µm.

The bone’s lacunae were segmented from the SRµCT images using Dragonfly (Fig. 6). The lacunae were carefully segmented by applying a simple threshold, which segmented all the voids within the bone. These segmented voids were then filtered based on size, requiring their volumes to be between 65 and 700 µm^3^. To reduce the noise contribution to the segmentation, the segmentation components were required to touch a second segmentation performed by applying a median filter (k = 3) and then simple thresholding. Incomplete lacunae that touched either end of the scan were removed to ensure only complete lacunae were measured. To measure individual lacunae, the segmentation was converted to individual components using connected components (26-connected). These were reduced to components with an aspect ratio $\ge$ 0.05 to exclude long, thin, rod-like components that occur due to artifacts (Fig. 6g). The segmentation of the lacunae was further corrected by manually removing components that were incorrectly segmented due to noise contribution.

The canals within the bone were segmented from the SRµCT images using Dragonfly. The canals were segmented by applying two sequential median filters to the image (k = 3). With the filtered image, the canals were segmented through simple thresholding and subtraction from the background around the bone. The canal segmentation was further refined by requiring the canals to have a volume of at least 750 µm^3^.

For each sample, the canal segmentation was combined with the segmentation of the endosteal and periosteal edges of the bone to describe the bone’s vasculature. The distance from the vasculature segmentation and its components was computed using Dragonfly. This distance map determined the minimum distance to the vasculature for each lacuna.

An average mineralization profile for bone surrounding the lacunae was constructed for each sample. The distance from the lacunae segmentation was computed to create these profiles using Dragonfly. These distances were binned by multiples of the voxel spacing (1.3 µm). To generate the mineralization profiles, these distance maps were combined with the mineralization information in the SRµCT images that were reconstructed with phase retrieval. A mineralization profile was constructed for each lacuna.

### Statistical analysis

Statistical analysis comparing four groups was performed using a two-way ANOVA with Tukey correction using GraphPad Prism8 (GraphPad Software, Inc). Comparisons between the two groups were analyzed using an unpaired two-tailed Student’s t-test. All outcome values are expressed as mean ± SD. Statistical significance is determined when p-values are less than or equal to 0.05, with sample size (N) described in each experiment and figure legend.

In instances of nested data where each bone sample contains repeated measures, a mixed model was used to perform statistical comparisons^45^. This study used a mixed model to analyze lacunar measurements, such as lacunar volume, mineralization, and distance to the vasculature. In these variables, a measurement is associated with each lacuna, which belongs to a specific bone. This nested data schema is well-suited for statistical comparisons with a mixed model. To perform statistical comparisons on the mineralization profiles, TMD values were binned by distance and compared using a mixed model. These p-values were then corrected with the Hochberg procedure to determine if there was a significant difference^46^. To determine the distance values where mineralization was significantly different, the Benjamini-Hochberg p-value adjustment was applied to control for false discovery rate^47^.

### Supplementary Information

Supplementary Information.

Supplementary Video 1.

## Data Availability

Data supporting this study’s findings are available from the corresponding author upon reasonable request.
