# Supplementary material for: Spatial control of perilacunar canalicular remodeling during lactation
Source: Sci Rep. 2024 Jun 25;14:14655. doi: 10.1038/s41598-024-63645-0 (PMC11199490; doi:10.1038/s41598-024-63645-0)
Supplement: Supplementary file 1 — Supplementary Information. [file 41598_2024_63645_MOESM1_ESM.pdf]

Spatial control of perilacunar canalicular remodeling during lactation
Supplemental Information

Michael Sieverts[1], Cristal Yee[2], Minali Nemani[2], Dilworth Y. Parkinson[3], Tamara Alliston[2], and Claire Acevedo*[1,4, 5]

[1] *Department of Mechanical Engineering, University of Utah, Salt Lake City, UT, 84112, USA*
[2] *Department of Orthopedic Surgery, University of California, San Francisco, CA 94131, USA*
[3] *Advanced Light Source, Lawrence Berkeley Laboratory, Berkeley, CA, 94720, USA*
[4] *Department of Biomedical Engineering, University of Utah, Salt Lake City, UT, 84112, USA*
[5] *Department of Mechanical and Aerospace Engineering, University of California San Diego, San Diego, CA, 92161, USA*

Overview

This document contains the details and results related to:

- Quantitative RT-qPCR
- Immunofluorescence
- Standard Micro-computed Tomography Measurements
- Synchrotron Radiation Micro-computed Tomography Measurements
- Syris Phase Simulation

*Corresponding author. Email: claire.acevedo@gmail.com

Quantitative RT-qPCR

Gene expression data were obtained from harvested humeri bone from 16-week-old mice, cleaned of any muscle and periosteum. The epiphysis was removed, and the bone marrow was removed via centrifugation. Humeri bones were then snap-frozen in liquid nitrogen and homogenized with an Omni homogenizer in Qiazol Lysis Reagent (Qiagen, Cat # 79306) as previously described [1, 2, 3]. RNA isolation was performed using the RNAeasy Mini Kit (Qiagen, Cat # 74106) following the manufacturer's instructions. Quantification of the extracted RNA was obtained using a Nanodrop spectrophotometer (Thermo Fisher Scientific), and 3ug of RNA was reversed-transcribed using an iScript cDNA synthesis kit (BioRad, Cat # 1708891) following the manufacturer's instruction. For reverse transcription quantitative real-time PCR (RT-qPCR) reactions, 60ng of cDNA from n=6-8 mice/group was performed using the primer sequences in Table S1. Technical replicates were used in each sample (n=6-8 mice/group). Analysis of RT-qPCR was quantified using the comparative C(T) method [4, 5], also known as the DDCt method, to obtain the fold change and normalized to the housekeeping gene, beta-actin.

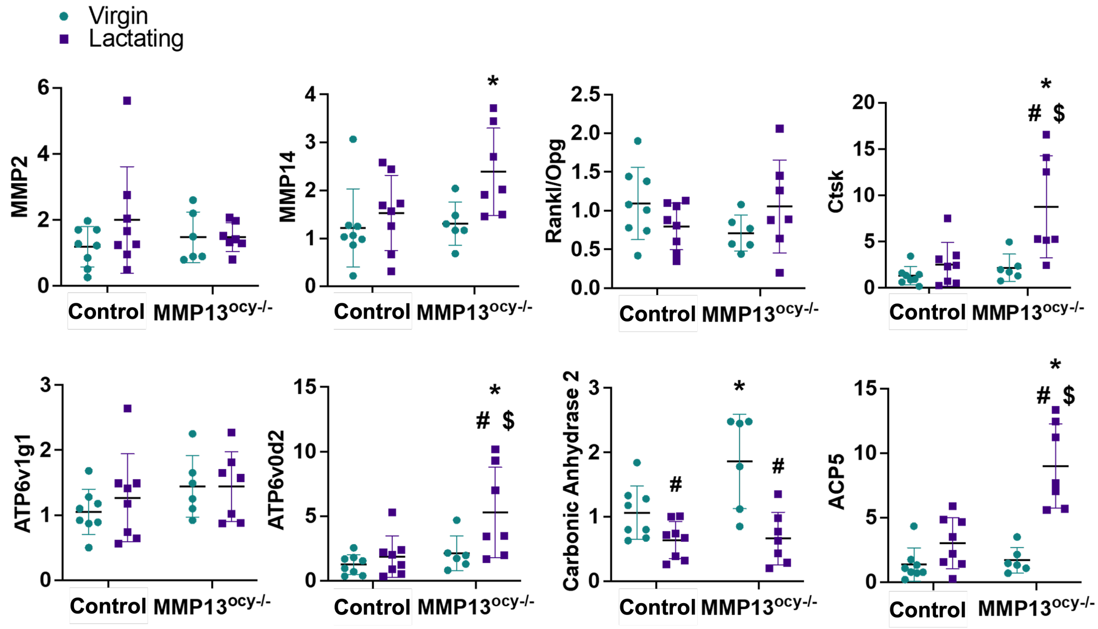

Figure S1: PLR-related mRNA expression (MMP2, MMP14, Rankl/Opg, Cathepsin K (Ctsk), ATP6v1g1, ATP6v0d2, Carbonic Andhyrase 2, ACP5 (tartrate-resistant acid phosphatase 5, TRAP)) was analyzed from osteocyte-enriched bone (free from marrow, periosteum, and trabeculae) and represented as fold change. Fold changes of mRNA expression graphs display mean and error bars indicated SD determined from two-way ANOVA *$p<0.05$ compared to Virgin WT, #$p<0.05$ compared to Virgin MMP13$^{ocy-/-}$, and \$$p<0.05$ compared to Lactating WT by Two-Way ANOVA (n=6-8 mice/group).

Table S1: Primer sequences for RT-qPCR analysis

Gene	Accession Number	Forward Primer	Reverse Primer	Product length
Acp5 (tartrate resistant acid phosphatase 5, TRAP)	NM_001102405	5’-CGTCTCTGCACAGATTGCAT-3’	5’-AAGCGCAAACGGTAGTAAGG-3’	75
Atp6v0d2	NM_175406	5'-TCTTGAGTTTGAGGCCGACAG-3’	5'-GCAACCCCTCTGGATAGAGC-3’	125
Atp6v1g1	NM_024173	5'-CCGTTCTCTCAGCCCAAAGT-3'	5'-CTCCGGTTCTTTCGCTTGC-3'	125
Beta actin	NM_007393	5'-CTCTGGCTCCTAGCACCATGAAGA-3'	5'-GTAAAACGCAGCTCAGTAACAGTCCG-3'	200
Carbonic anhydrase 2	NM_009801	5'-GAGCTTCACTTGGTTCACTGG-3'	5'-TGTGAGGCAGGTCCAATCTTC-3'	113
Ctsk (cathepsin K)	NM_007802.4	5’-GAGGGCCAACTCAAGAAGAA-3’	5’-GCCGTGGCGTTATACATACA-3’	203
Mmp13	NM_008607.2	5’-CGGGAATCCTGAAGAAGTCTACA-3’	5’-CTAAGCCAAAGAAAGATTGCATTTC-3’	75
Mmp14	NM_008608.4	5’-AGGAGACGGAGGTGATCATCATTG-3’	5’-GTCCCATGGCGTCTGAAGA-3’	142
Mmp2	NM_008610.3	5’-AACGGTCGGGAATACAGCAG-3’	5’-GTAAACAAGGCTTCATGGGG-3’	125

Immunofluorescence

Right femurs of 16-week-old mice were dissected, fixed in 10% neutral buffered formalin (NBF), decalcified in 10% disodium and tetrasodium EDTA, followed by serial ethanol dehydration and paraffin-embedded. Paraffin sections (6µm) in the axial plane across the mid-cortical region of the femur were stained with primary antibody for MMP13 (1:20, ab39012) with Alexa Fluor 594 Tyramide SuperBoost Kit (Invitrogen, Cat #: B40925) following the manufacturer's instructions. Briefly, paraffin sections were deparaffinized, rehydrated, and incubated in Uni-Trieve (Innovex Biosciences, Cat #: NB325) at 65°C for 30 mins, blocked with 10% goat serum for 1hr at room temperature, and incubated with primary antibody for MMP13 or Rabbit IgG (ab172730) as a negative control for overnight at room temperature. Secondary goat anti-rabbit IgG (provided in the Alexa Fluor 594 Tyramide SuperBoost Kit) was incubated for 1-2 hrs at room temperature and detected using Alexa Fluor Tyramide working solution for 10 mins at room temperature. Slides were mounted with Prolong Gold antifade Mountant with DAPI (Invitrogen, Cat #: P36935) and imaged using Leica DM2500. Four regions of each 2-4 sections were imaged at 40X from every n=2-3 mice/group.

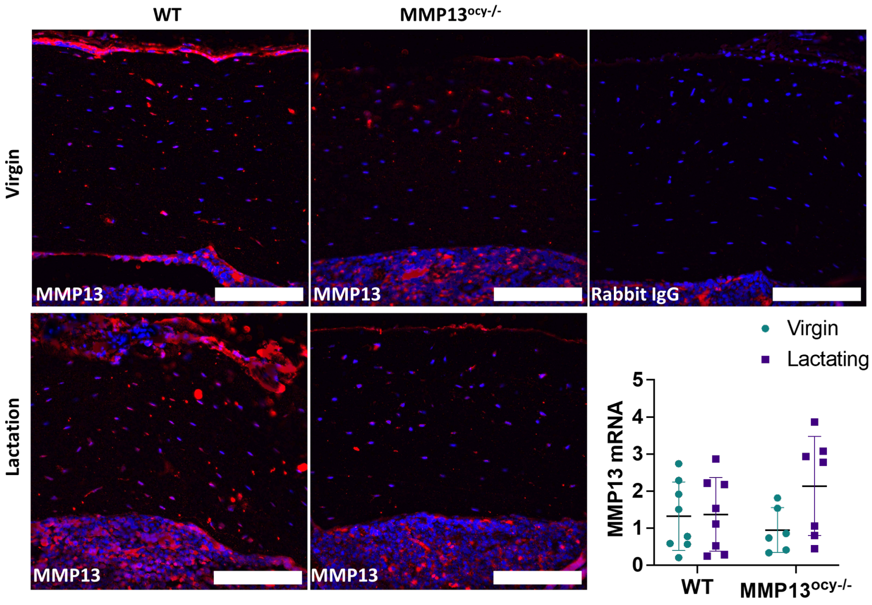

Figure S2: Immunofluorescence (IF) on cortical bone in the mid-diaphysis femur from 16-week-old virgin and lactating WT and MMP13$^{ocy\text{-}/\text{-}}$ mice revealed reduced MMP13 protein expression (n=2-3 mice/group) in virgin MMP13$^{ocy\text{-}/\text{-}}$ compared to virgin WT controls (scale bar: 100um). Virgin MMP13$^{ocy\text{-}/\text{-}}$ osteocyte-enriched humeri had a modest 28% decrease in MMP13 mRNA expression (n=6-8 mice/group). Rabbit IgG was used as a negative control in IF stain (Scale bar: 100um). Graph of MMP13 mRNA display fold change with mean and SD determined with Two-Way ANOVA (n=6-8 mice/group).

Standard Micro-computed Tomography Measurements

Table S2: MicroCT of 16-week-old female (virgin and lactating) WT and MMP13$^{ocy-/-}$ mouse bones.

	WT (n=7)	WT Lactation (n=7)	MMP13$^{ocy-/-}$(n=7)	MMP13$^{ocy-/-}$ Lactation (n=7)
**Distal Femur**				
Tb. TV (mm³)	1.69 ± 0.12	1.92 ± 0.13*#	1.55 ± 0.15	1.92 ± 0.17*#
Tb. BV (mm³)	0.17 ± 0.05	0.19 ± 0.04#	0.34 ± 0.13*	0.25 ± 0.07
Tb. BV/TV (%)	0.10 ± 0.03	0.10 ± 0.02#	0.21 ± 0.07*	0.13 ± 0.04#
Tb. Conn.D (1/mm³)	126.90 ± 60.79	264.00 ± 58.58	272.15 ± 28.53	507.74 ± 196.06*#$
Tb. SMI	2.50 ± 0.40	2.46 ± 0.18#	1.34 ± 0.60*	2.22 ± 0.50#
Tb. N (1/mm)	4.05 ± 0.47	4.93 ± 0.34	5.59 ± 0.63*	6.35 ± 0.89*$
Tb. Th (mm)	0.04 ± 0.004	0.03 ± 0.003*#	0.05 ± 0.005	0.03 ± 0.005*#
Tb. Sp (mm)	0.25 ± 0.03	0.21 ± 0.01*	0.17 ± 0.03*	0.16 ± 0.03*$
Tb. BMD (mg HA/ccm)	146.29 ± 30.44	146.91 ± 18.64#	276.73 ± 73.26*	195.10 ± 36.88#
Tb. TMD (mg Ha/ccm)	1076.43 ± 24.07	964.51 ± 22.79*#	1057.38 ± 15.42	915.94 ± 33.51*#$
**Midshaft Femur**				
Ct. TV (mm³)	1.63 ± 0.10	1.63 ± 0.10	1.54 ± 0.12	1.59 ± 0.08
Ct. BV (mm³)	0.81 ± 0.05	0.63 ± 0.03*#	0.85 ± 0.07	0.65 ± 0.09*#
Ct. BV/TV (%)	0.50 ± 0.01	0.39 ± 0.02*#	0.56 ± 0.04*	0.41 ± 0.07*#
Ct. SMI	0.37 ± 0.01	0.24 ± 0.11	-0.02 ± 1.02	0.38 ± 0.72
Ct. Th (mm)	0.21 ± 0.01	0.16 ± 0.01*#	0.23 ± 0.02	0.15 ± 0.03*#
Ct. BMD (mg HA/ccm)	722.16 ± 16.23	555.06 ± 30.78*#	805.24 ± 58.80	593.54 ± 94.32*#
Ct. TMD (mg Ha/ccm)	1415.64 ± 18.61	1389.57 ± 17.15	1413.63 ± 20.37	1402.45 ± 26.84

Data represents mean ± standard deviation for parameters measured. Trabecular (Tb.) and cortical (Ct.) bone was characterized by measuring total volume (TV), bone volume (BV), bone volume fraction (BV/TV); connectivity density (Conn. D.), structural model index (SMI), number (N), thickness (Th), separation (Sp), bone mineral density (BMD), and tissue mineral density (TMD).

*$p<0.05$ compared to WT virgin
#$p<0.05$ compared to MMP13$^{ocy-/-}$ virgin
$$p<0.05$ compared to WT lactation

Synchrotron Radiation Micro-computed Tomography Measurements

To further examine bone resorption due to lactation, we measured the endocortical perimeter (Ec.Pm) and the periosteal perimeter (Ps.Pm) for each bone using Dragonlfy's Bone Analysis Tool. Due to lactation, we observed a 16% increase Ec.Pm in the control (WT) group (p=0.18) and a significant 31% increase in Ec.Pm in the MMP13$^{ocy-/-}$ group. No significant changes were detected in the Ps.Pm as a result of lactation. These results confirm that cortical bone resorption during lactation occurs at the bone's endocortical region [6].

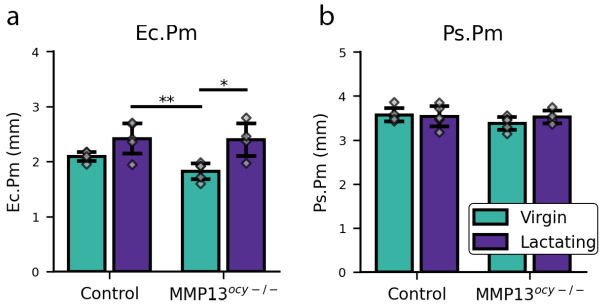

Figure S3: Measurements of cortical tibia bone surface perimeter using SRµCT images. a) Measurements of the endocortical perimeter (Ec.Pm). b) Measurements of the periosteal perimeter (Ps.Pm).

Syris Phase Simulation

During the reconstruction of the synchrotron X-ray radiation micro-computed tomography (SRµCT) images, we utilized phase retrieval to retrieve the absorption component of the image and minimize the impact of phase at material interfaces. The effectiveness of the phase retrieval was confirmed through phase simulation using Syris, the synchrotron radiation imaging simulation repository [7] (Figure S4). To simulate the phase contribution in bone, we first took a reconstructed SRµCT image and binarized it using simple thresholding. Using the binarized image, we obtained image projections using Tomopy [8]. Phase was simulated in these projections using Syris. Before phase propagation, each projection was converted to a static body that would mimic the material properties of hydroxyapatite using Syris. These projections with a phase contribution were then reconstructed using Tomopy. Images were reconstructed with and without phase retrieval to analyze the effectiveness of phase retrieval in reducing the phase component of the image. Paganin phase retrieval was performed using the phaseCT repository from Forien et al. [9, 10]. The absorption (β) and phase (δ) coefficients for hydroxyapatite were used for this phase retrieval. The β and δ values used were 1.48130805E-08 and 2.01842317E-06, respectively. We found that Syris closely modeled the real phase contribution in bone and that the Paganin phase retrieval implemented by Forien et al. [9, 10] effectively minimized this phase contribution. Due to these results, we performed localized mineralization measurements in the bone surrounding the lacunae.

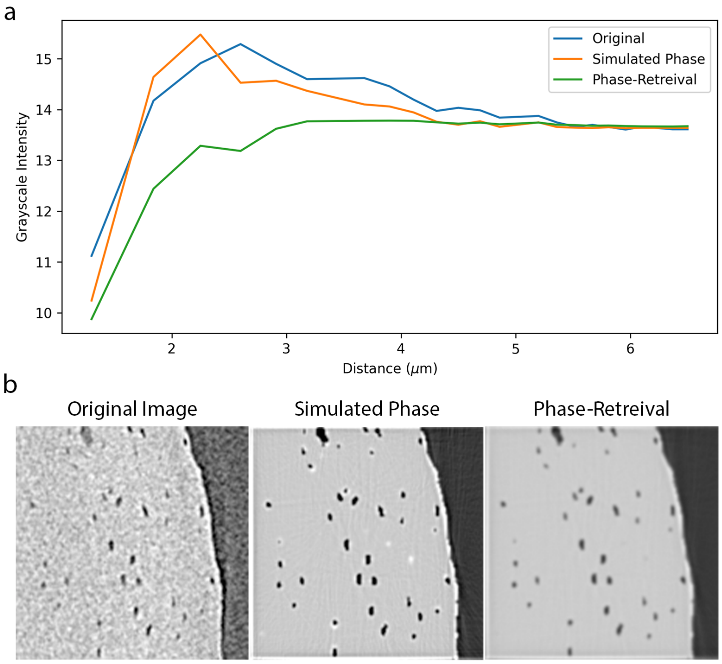

Figure S4: Phase simulation results using Syris. a) Mineral profiles computed using a 3D distance map away from the lacunae for the original image, the simulated phase image, and the simulated phase retrieval image. b) Representative image slices showing the original image, the simulated phase image, and the simulated phase retrieval image.

References

[1] Neha S Dole et al. "Osteocyte-intrinsic TGF-β signaling regulates bone quality through perilacunar/canalicular remodeling". In: *Cell reports* 21.9 (2017), pp. 2585–2596.

[2] Courtney M Mazur et al. "Osteocyte dysfunction promotes osteoarthritis through MMP13-dependent suppression of subchondral bone homeostasis". In: *Bone research* 7.1 (2019), pp. 1–17.

[3] Tristan W Fowler et al. "Glucocorticoid suppression of osteocyte perilacunar remodeling is associated with subchondral bone degeneration in osteonecrosis". In: *Scientific reports* 7.1 (2017), pp. 1–13.

[4] Thomas D Schmittgen and Kenneth J Livak. "Analyzing real-time PCR data by the comparative CT method". In: *Nature protocols* 3.6 (2008), pp. 1101–1108.

[5] Kenneth J Livak and Thomas D Schmittgen. "Analysis of relative gene expression data using real-time quantitative PCR and the 2- $\Delta\Delta$CT method". In: *methods* 25.4 (2001), pp. 402–408.

[6] Simon Y Tang et al. "Matrix metalloproteinase–13 is required for osteocytic perilacunar remodeling and maintains bone fracture resistance". In: *Journal of Bone and Mineral Research* 27.9 (2012), pp. 1936–1950.

[7] Tomáš Faragó et al. "syris: a flexible and efficient framework for X-ray imaging experiments simulation". In: *Journal of Synchrotron Radiation* 24.6 (2017), pp. 1283–1295.

[8] Doga Gürsoy et al. "TomoPy: a framework for the analysis of synchrotron tomographic data". In: *Journal of synchrotron radiation* 21.5 (2014), pp. 1188–1193.

[9] David Paganin et al. "Simultaneous phase and amplitude extraction from a single defocused image of a homogeneous object". In: *Journal of microscopy* 206.1 (2002), pp. 33–40.

[10] Jean-Baptiste Forien and K Aditya Mohan. *PhaseCT*. `https://github.com/jbforien/phaseCT`. 2021.
